# Detection and Classification of Artifact Distortions in Optical Motion Capture Sequences

**DOI:** 10.3390/s22114076

**Published:** 2022-05-27

**Authors:** Przemysław Skurowski, Magdalena Pawlyta

**Affiliations:** 1 Department of Graphics, Computer Vision and Digital Systems, Faculty of Automatic Control, Electronics and Computer Science, Silesian University of Technology, Akademicka 16, 44-100 Gliwice, Poland; 2 Polish-Japanese Academy of Information Technology, Koszykowa 86, 02-008 Warsaw, Poland

**Keywords:** motion capture, artifact classification, artifact detection, reconstruction, anomaly detection

## Abstract

Optical motion capture systems are prone to errors connected to marker recognition (e.g., occlusion, leaving the scene, or mislabeling). These errors are then corrected in the software, but the process is not perfect, resulting in artifact distortions. In this article, we examine four existing types of artifacts and propose a method for detection and classification of the distortions. The algorithm is based on the derivative analysis, low-pass filtering, mathematical morphology, and loose predictor. The tests involved multiple simulations using synthetically-distorted sequences, performance comparisons to human operators (concerning real life data), and an applicability analysis for the distortion removal.

## 1. Introduction

Motion capture (mocap) systems [1,2] play important roles in modern computer graphics, where they are applied in gaming and movie FX to generate realistic character animations. Prominent applications of mocap systems could also be found in biomechanics and medical sciences [3]. To date, the most reliable technology is the marker-based optical mocap (OMC)—it is known as the ‘gold standard’ as it outperforms other mocap technologies. It utilizes visual tracking of active or retro-reflective passive markers. Trajectories of these markers are then used for animation of associated skeletons, which are used as key models in the animation of human-like or animal characters.

The process of acquiring marker locations is error prone. Distortions occurring in the mocap sequences can be simply divided into two classes—random noise and algorithmically-introduced artifact distortions. Random noise is a consequence of the stochastic processes resulting in different kinds of distortions in a mocap sequence. It has been studied in numerous works [4,5,6,7]. Among the types of noise, the most prominent [8] is white noise, which can be efficiently filtered out [9] or ‘smoothed’. Numerous methods have been proposed [10] utilizing low-pass filtering, interpolating methods, or moving averages.

Artifact distortions are introduced by reconstruction algorithms present in mocap pipelines; they could be regarded as momentary systematic errors. These distortions introduce trajectory modifications of different appearances and of larger amplitudes. At higher levels of mocap processing, when the marker motion is remapped to drive the skeleton animation [11], the false positions of markers result in erroneous poses, which degrade the animation or biomechanical measurements. All distortions, gaps, and artifacts occur commonly in mocap sequences, influencing the praxis of a mocap operator. Since trajectory ’mis’-shapes are poorly filtered out by simple noise removing algorithms, standard industrial quality processing of mocap sequences require visual trajectory screenings and manual trajectory editing by operators. It is a painstaking process that could be assisted with software support for trajectory reconstruction; however, these capabilities are limited, and these methods could also degrade the results if used improperly.

Despite the common knowledge about artifact problems, this topic has not been fully recognized. In related works, researchers have mainly focused on error prevention during gap reconstruction, focusing on the efficiency of error removal (including artifacts) in terms of root mean squared error (RMSE). To our knowledge, our proposal is novel as it identifies erroneous intervals and classifies them accordingly.

The key motivation regarding the development of distortion classifiers is that, for each different distortion class, we can select an appropriate method of suppressing (e.g., for the rectangular distortion, which is a result of mistaken marker labeling—it would be enough to find its counterpart marker and swap erroneous parts of the trajectories to achieve perfect reconstruction).

In the article, we propose a marker-wise method for detection and classification of systematic errors—artifacts. The proposed approach is skeleton-free; therefore, it is able to adapt to virtually any vertebrate subject. There are two basic assumptions: the rigid body model and a correlation of marker trajectories. A rigid body model was assumed for the functional body mesh (structured point cloud) [12], which we used to represent the subject’s body hierarchy. The next assumption stems from the former—it is the fact that the movements of markers are highly correlated and predictable when they are placed on common body parts (e.g., limbs). We employed a deviation of a trajectory from the prediction as a criterion for classification.

The proposed method is intended to support the mocap operator. It could be used in various ways, either assisting the operator by pointing out potential artifacts, or as a fully automatic method (combined with filtering and capable of detecting and removing artifacts). Our results show that the detection efficiency is on par with operators with intermediate experience, and it outperforms novice ones. Both approaches were verified in the experiments: E2—where we compared recognition abilities to the human operators and E3—where we verified recognition combined with several reconstruction methods.

The article is organized as follows: in Section 2, we disclose the background for the article—the mocap pipeline with distortion sources and former works on the distortions in optical mocap systems; Section 3 describes the proposed method with its rationales and design considerations. In Section 4, we test the method for its performance and discuss the results. Section 5 summarizes the article.

## 2. Background

### 2.1. Sources and Types of Distortions

There are two main sources of artifacts occurring in the markers in optical motion capture signals: software-caused (the main scope of the article) and soft tissue-caused artifacts (e.g., [13]). A soft tissue-caused artifact represented the actual marker motion; however, the marker was moved relative to the underlying bone because of local skin deformations.

**Figure 1:**
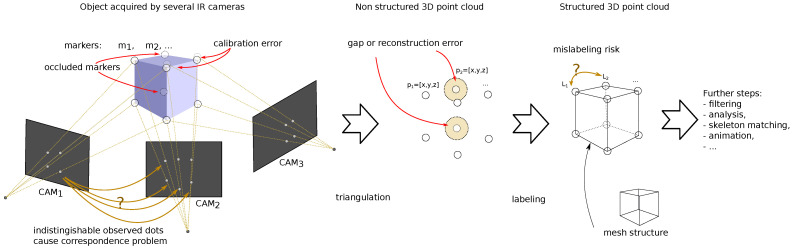
Processing in the early stages of the motion capture pipeline with distortion sources (red) and problems to solve (yellow), question marks (?) indicate ambiguous choices.

In optical mocap, marker tracking is obtained by the image registration of the marker position by multiple IR cameras. A multi-view observation allows for the reconstruction of marker trajectories (3D positions over time) through the triangulation of 2D position recordings, which are registered by multiple cameras. The process is error-prone and various sources of the distortions can be identified, as depicted in Figure 1. Besides conventional stochastic noise, two main error sources in marker registration are gaps and erroneous marker matching. Gaps occur when the marker disappears from the camera view, due to locating the body part outside the scene (camera range), covering markers with another body part (occlusion). In such cases, reconstruction algorithms can be sources of errors. Marker matching occurs twice in the mocap pipeline. First, prior to the triangulation, it is necessary to perform marker matching in multiple 2D views of a single frame to identify corresponding 2D locations of markers. Another marker matching procedure is labeling (naming); it is performed among the different frames, where it is necessary to identify corresponding successive positions of 3D markers in the sequences of the frames. These software procedures can result in one trivial and four regular types of distortions, which can be observed in current mocap systems. These are:

1. Simple gap—appears when reconstruction algorithms give up, it is the least type of concern (a trivial case);
2. Single peak—caused by transient erroneous marker matching techniques. It is simple to detect;
3. Heavy noise of a much larger amplitude than ordinary noise introduced by frequent erroneous marker matching techniques;
4. Rectangular distortion—forward (followed by backward) steps caused by mismatching the 3D positions of the markers (part of the 3D trajectory is assigned to another marker) or due to the erroneous marker reconstruction based on a rigid body model;
5. Slow value change—two potential sources—accumulated reconstruction errors in successive frames (e.g., when there is deformation of a body, which is the failure of a commonly assumed rigid body model) or the result of low-pass filtering of peaks.

**Figure 2:**
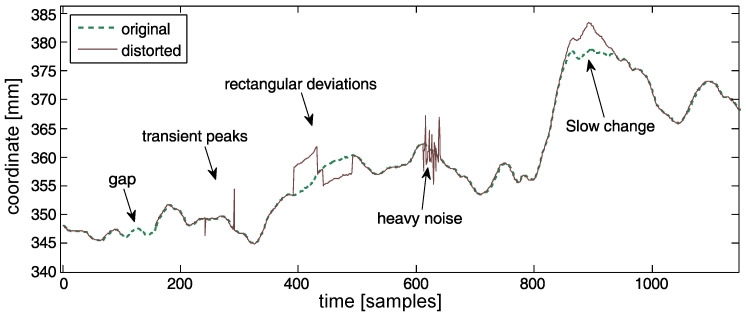
Identified types of distortions inpainted into exemplary data—the first coordinate of the first marker (head) of the IM subject.

All of the above classes are depicted in Figure 2. They can be roughly divided into two basic classes—sudden (2–4) and slow (5) changes to the trajectory. It is worth noting that, aside from software sources, soft tissue over the skeleton is an additional distortion source as it denies the rigid body model. Usually, these artifacts take ‘slow change’ forms, so they fit into the above classification.

### 2.2. Previous Work

To the authors’ knowledge, this work is the first proposal for the identification and classification of artifact distortions in mocap sequences. Of course, the knowledge of reconstruction imperfections and artifacts is present in wider backgrounds of mocap technology and research; artifacts are referenced to in the former works as errors.

Most research studies on mocap signal processing mainly focus on preventing the occurrence of artifacts. Therefore, works on motion capture areas are related mostly to the occlusion gap filling problem and are oriented toward minimizing the gap filling error. They involve various methods for signal reconstruction, when the marker is lost in a recorded sequence. Approaches include interpolation [14], fusion of weak regressors [15], inverse kinematics [16], skeletal model [17], and inter-marker correlations [18,19]. Nowadays, deep neural networks are hot topics) [20,21,22]. However, such approaches usually require a lot of training data, which might not be available—every new marker configuration, new type of activity, or even individual actor might require retraining the network. It might be difficult, especially for the deep NNs, which may require a lot of training examples; therefore, deep NNs might just be feasible for typical situations.

The methods differ in the assumptions, performances, and complexities; some assume constraints from rigid body constraints and others employ skeletal models (or nothing). Constraint efficiency depends on how adequate and accurate the assumed model is. There could be discrepancies between the rigid body and modeled body segments [23], on the other hand, skeleton-based methods are sensitive to the accurate estimations of model parameters—bone lengths and marker placements—with respect to the underlying bone.

Typically, the errors in reconstruction methods appear in slow changes, which in some cases [17] are elongated so much that they appear as constant biases. The main factor that decides whether a certain method is suitable or not is the length of the gap. Simple signal-based methods (e.g., interpolation) work well for short errors, whereas complex model-based methods are better suited for long gaps.

The other approach, resulting in error/artifact reduction in the mocap pipeline, takes certain stages of the pipeline into consideration. Researchers have focused on partial problems in the motion capture pipeline, and they perfected these individual steps, improving the system configuration [24], e.g., the number and layout of the cameras, calibration [25], and labeling [26,27,28,29]. Such approaches undoubtedly reduce errors and improve the overall performance of the mocap pipeline, yet they are not perfect. Errors of reconstruction still occur; therefore, there remains room for improvement.

We identified only one research proposal slightly similar to ours, where erroneous intervals were explicitly identified for further cleaning. In [17], actual markers drove the skeleton first, then virtual markers were placed onto virtual skin. The positions of the actual and virtual markers were compared; if the marker positions did not match, such intervals were assumed to be erroneous and filtered; however, no identifications of distortion types were used.

## 3. The Proposal

### 3.1. Premises—Correlation of Trajectory Coordinates

The correlation between the locations and gradients of markers allowed us to propose a method to classify all types of distortions. Since the variables in mocap sequences can be strongly (positively and negatively) correlated within the groups, artifacts introduced by reconstruction algorithms should differ significantly enough to distinguish them on the basis of the proper trajectories of neighboring markers.

**Figure 3:**
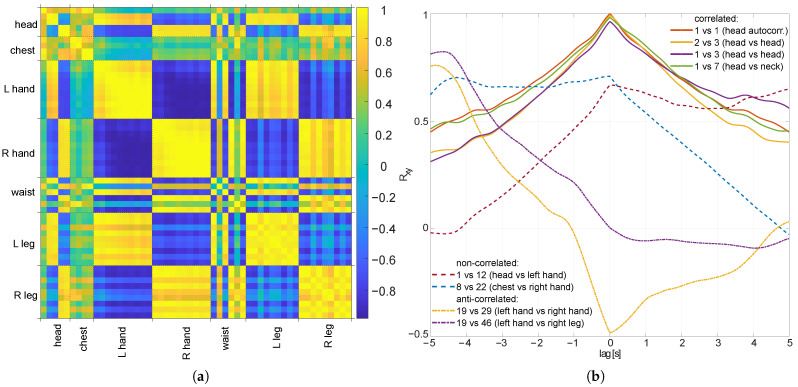
Correlation between X position of markers in exemplary sequence (fast walking HJ subject): (**a**) the whole sequence, all 53 markers; (**b**) inter-marker correlation function for the selected correlated and non-correlated markers.

In Figure 3a, we present a correlation coefficient (CC) in the form of a distance matrix. It demonstrates structural dependencies in the correlations between the marker positions. One can easily observe the clusters formed by the body parts—hands, torso, legs, and so on. The correlation is high within individual body parts (both positive and negative); on the other hand, the correlates between body parts depend on the registered motions—in case of natural walking, the hand positions would counter-correlate, whereas with a butterfly swimming motion, the hand positions would correlate.

The time aspect of the correlation is depicted in Figure 3b; it presents the correlation and autocorrelation functions for several marker pairs. It clearly illustrates the correlation between successive marker locations and between locations of the markers located in the common body segments and connected body segments (e.g., head and neck).

To conclude this line of reasoning, we suggest that we could reliably identify the outstanding markers on the basis of the markers correlated within groups—from the common body segment or parent body part.

### 3.2. The Method Overview

The proposal is feasible thanks to the data correlations in mocap sequences, which make the predictions feasible and allow for reliable estimations of the actual positions of markers.

**Figure 4:**
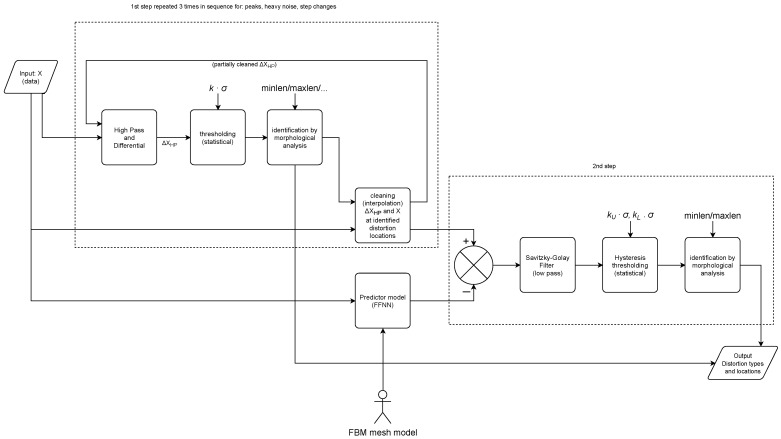
Conceptual schematic of the proposed algorithm.

The key idea of the algorithm is to use model (prediction) results as a verification criterion for the data. If the data fall too far from the prediction results, then they are rationally considered distortion, and could be assigned into a distortion class using a pattern recognition method. Each distortion class is identified at a separate stage; it is cleaned from the signal using interpolation. The signal is then passed to the next stage of detection, from the simple distortion (single peaks) to the most difficult (slow changes). The conceptual scheme of the proposed method is depicted in Figure 4; for a detailed view, please refer to the unfolded schematic of the processing pipeline given in Section 3.4.

The method for anomaly identification and classification depends on the type of distortion. Sudden changes to the trajectory are identified on the basis of the differential signals and low-pass filters (as predicting models) with stats-based thresholding and mathematical morphology. This allows distinguishing between the types of sudden changes. Slow change detection is based on the hysteresis thresholding of residuals with backward regrowing of identified segments.

Three predictive models were employed in our pipeline. In case of sudden change detection, which is the simple case, we moved median and Savitzky–Golay filters to identify legitimate changes in the signal. To identify short-term distortions, we employed median filters (as they are estimator-resistant to peak changes). For longer term distortions, we employed Savitzky–Golay, which could follow a low-pass signal waveform in the presence of a high-frequency noise. For the detection of slow changes, we employed neighbor-based predictors—initially we assumed a polynomial predictor based on the least squares method, which we gave up in favor of a feed-forward neural network (FFNN), yet we decided to include it in the description, as it depicts the development process.

### 3.3. Regressive Models

The efficiency of the overall approach depends on the quality of the model predictors and the ability to approximate the real location of a given marker (the location where it should be), on the basis of its own or neighboring markers locations from past, present, or future. In general, the regression model (predictor) [30] has the form of the function:

(1) $$\hat{Y_{i}}=f\left(X_{i},\beta_{i}\right)+R_{i},$$

which in the linear [31] case yields:

(2) $$\hat{Y_{i}}=X_{i}\beta_{i}+R_{i},$$

where for *N* observations of *M* regressor variables, $\hat{Y_{i}}$ is the *N*-element long column of the predicted values of the *i*-th variable, $X_{i}$ is the *N*-by-*M* design matrix for the model, $\beta_{i}$ is the *N*-element column vector of coefficients, $R_{i}$ is the error (residual). The model coefficients are estimated on the basis of $X_{i}$ and $Y_{i}$, a column vector of goal values with the least squares method (LSM) is denoted as:

(3) $$\beta_{i}={\left(X_{i}^{\prime }X_{i}\right)}^{-1}X_{i}^{\prime }Y_{i}.$$

The residual is the remaining value, which is the non-predicted/non-correlated part of the signal, given simply as:

(4) $$R_{i}=Y_{i}-\hat{Y_{i}}.$$

The part that will be further analyzed is the residual. Its probability follows the Laplace (double exponential) distribution:

(5) $$f\left(x|\mu ,b\right)=\frac{1}{2b}e^{\left(-\frac{\left|x-\mu \right|}{b}\right)},$$

where: μ is the mean value equal to zero in our case, *b* is a dispersion parameter calculated on the standard deviation as $b=\sigma /\sqrt{2}$. Therefore, the standard deviation of the residual (denoted as $\sigma_{R}$) can be employed to evaluate the quality of the prediction; moreover, it indicates that such a centered distribution allows for an efficient outlier detection using the thresholding based on the standard deviation (e.g., three-sigma rule).

#### 3.3.1. Savitzky–Golay Filter

Savitzky–Golay filter [32] is a smoothing filter that is based conceptually on polynomial fitting in the least squares sense (there are efficient convolution-based implementations). Its output is a value of the polynomial function fit locally to the data. The coefficients ($c^{l}$) of the *L*-th order are fit to the data within the sliding window of size *M* centered around x(i); the filter output is the polynomial value for the midpoint. In the basic variant, the filter is ‘low-pass’, but a ‘high-pass’ can be obtained by a simple difference between the signal and its smoothed variant:

(6) $$\begin{array}{cc} p_{LP}\left(i\right) & =\sum_{l=0}^{L}c_{l}\cdot x^{l}\left(i\right) \end{array}$$

(7) $$\begin{array}{cc} p_{HP}\left(i\right) & =c\left(i\right)-p_{LP}\left(i\right) \end{array}$$

Its least squares design matrix can be simply noted as:

$$X_{i}=(\begin{array}{ccccc} 1 & x(i-M) & x^{2}\left(i-M\right) & \cdots & x^{L}\left(i-M\right)\\ \vdots & \vdots & \vdots & \ddots & \vdots \\ 1 & x\left(i+M\right) & x^{2}\left(i+M\right) & \cdots & x^{L}\left(i+M\right) \end{array}),$$

#### 3.3.2. Neighbor-Based Linear Least Squares Loose Model

Slow change detection requires the predictor to be able to avoid following slowly-accumulating changes in the signal; hence, we employed loose (weak) prediction, which does not rely on its own momentary positions of the marker, it only uses past and current positions of sibling and parent markers. Initially we employed the polynomial model; it was obtained with ordinary least squares (LS) and was conveniently planned using the Vandermonde matrix $X_{i}$, with some caution, as it could be ill-conditioned with growing polynomial orders, as:

$$X_{i}=\left(\begin{array}{cccccccc} 1 & x_{j}\left(1\right) & x_{j}^{2}\left(1\right) & \cdots & x_{j}^{L}\left(1\right) & x_{k}\left(1\right) & x_{k}^{2}\left(1\right) & \cdots \\ 1 & x_{j}\left(2\right) & x_{j}^{2}\left(2\right) & \cdots & x_{j}^{L}\left(2\right) & x_{k}\left(2\right) & x_{k}^{2}\left(2\right) & \cdots \\ \vdots & \vdots & \vdots & \ddots & \vdots & \vdots & \vdots & \cdots \\ 1 & x_{j}\left(N\right) & x_{j}^{2}\left(N\right) & \cdots & x_{j}^{L}\left(N\right) & x_{k}\left(N\right) & x_{k}^{2}\left(N\right) & \cdots \end{array}\right),$$

where: x(n) are successive 1 … *N* values of a single regressor variable, *L* is a polynomial order, i,j,k,⋯ are variable indices, such that i≠j,k,⋯.

Considering the predictor, the term order appears twice, meaning the context size—number of former values taken into prediction and polynomial order. Hence, to avoid confusion in the paper, we used the following notation for predictors and residuals

(8) $$P_{k}^{L}\left(i,n\right),\;R_{k}^{L}\left(i,n\right)$$

where: *L*—means polynomial order used in *X*, *k*—the number of past values of regressor variables used to construct *X*, *n*—the number (time) of frames in the sequence, *i* is the number of predicted variables.

**Figure 5:**
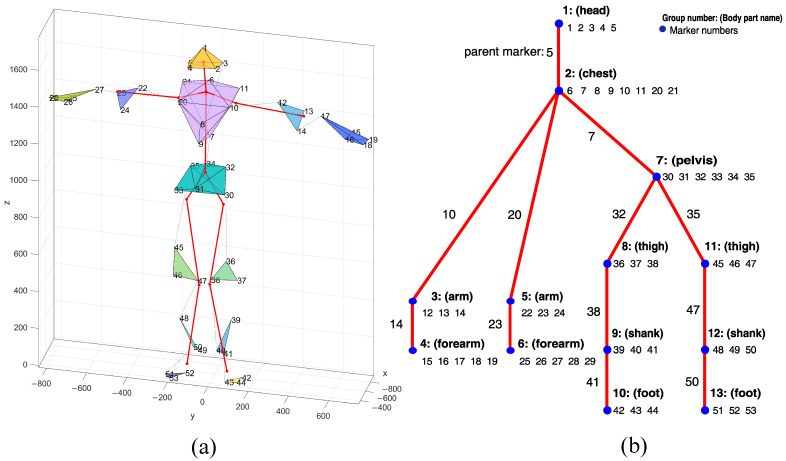
Outline of the body model, with body parts distinguished with individual random colors and underlying skeleton included (**a**), and corresponding parts hierarchy annotated with parents and siblings (**b**).

The selection of proper markers to formulate the predictor for each marker according to Equation (3) is based on the body’s hierarchical structure. For that purpose, we used a body structure as depicted in Figure 5a—a functional body mesh (FBM) [12] for an average human subject, inferred for the typical Vicon marker setup. The FBM hierarchy with the corresponding skeleton is shown in Figure 5b. The FBM represents a kinematic structure—group markers located on the structure of the body and a hierarchy as a tree of these groups. The structure-obtaining step must be performed for each class of subjects or different sets of markers separately. The design matrix *X* was composed of coordinates of parents and siblings in current and *k* former frames (but it excluded former coordinates of the considered marker). A parent cluster is represented by a single marker—the one that is closest in terms of gradient coherence and distance constancy.

**Figure 6:**
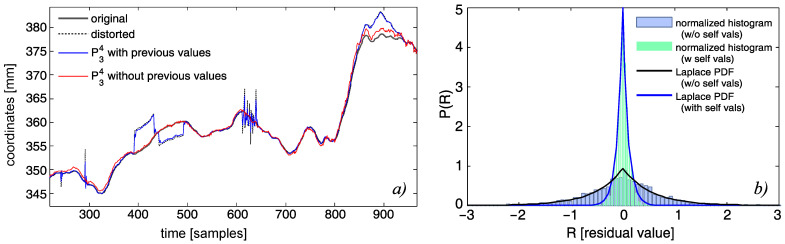
Performance of the two predictor models of $P_{3}^{4}$ (with and without former values of the predicted variables): (**a**) first dimension of the first marker (with artificial distortions); (**b**) residual histograms and corresponding Laplace PDFs for $R_{3}^{4}$ (for explanation of the model construction and parameters, see Equation (8)).

**Figure 7:**
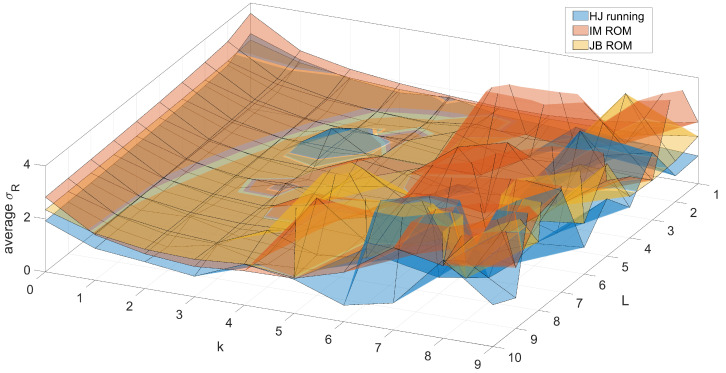
Predictor parameter tuning with preliminary data for three subjects, quality as standard deviation averaged over all markers; the tuned parameters: *L*—polynomial order, *k*—context size (lags).

Our demands toward the predictor were slightly specific. One obvious requirement was for it to be as precise as possible. Another (but contradictory) requirement was that it would not follow momentary changes induced by artifacts. Such requirements made us choose a special approach to formulate the *X* matrix; the predictor efficiency of the predictor depended on the data used. We neglected past values of considered markers—this was due to the fact that the largest correlation was between the current and past locations of a marker; therefore, it ensured the accuracy (Figure 6b). Unfortunately, in case of a distortion, it would make the predictor follow the artifact deviation; see Figure 6a. Next, we chose predictor parameters. Usually, the higher the degree of the polynomial and context size, the higher the precision; however, due to ill conditioning of *X*, it could reach a higher error with the growing polynomial order. Moreover, too large of an increase would not improve the predictor accuracy. The predictor parameters were tuned with numerical testing with preliminary data. We set up parameters to k=3 and L=4, as they appeared during the preliminary model tuning (Figure 7) to be a reasonable trade-off between the predictor accuracy and computation complexity.

Summarizing each row vector in *X* is long and is assembled of certain parts as given below:

(9) $$\begin{array}{cc} \; & \;X\left(n\right)=\left[\begin{array}{c} 1,\;\overset{\text{current}\;\text{value}\;\text{and}\;k\;\text{former}\;\text{of}\;\text{parent}\;\text{marker}\;(p)}{\overset{︷}{x_{p}\left(n\right),y_{p}\left(n\right),z_{p}\left(n\right),x_{p}\left(n-1\right),\cdots ,z_{p}\left(n-k\right)}}\;,\;\;\;\;\\ \;\;\;\;\overset{\text{current}\;\text{value}\;\text{and}\;k\;\text{former}\;\text{of}\;\text{first}..\text{last}\;\text{siblings}}{\overset{︷}{x_{s1}\left(n\right),y_{s1}\left(n\right),z_{s1}\left(n\right),x_{s1}\left(n-1\right),\cdots ,z_{slast}\left(n-k\right)}},\\ \vdots \\ \;\;\;\;\;\;\underset{\text{current}\;\text{value}\;\text{and}\;k\;\text{former}\;\text{of}\;\text{first}..\text{last}\;\text{siblings}\;\text{raised}\;\text{to}\;L\text{th}\;\text{power}}{\underset{︸}{x_{s1}^{L}\left(n\right),y_{s1}^{L}\left(n\right),z_{s1}^{L}\left(n\right),x_{s1}^{L}\left(n-1\right),\cdots ,z_{slast}^{L}\left(n-k\right)}} \end{array}\right]. \end{array}$$

We studied the polynomial models, thoroughly scanning the parameter space (see Figure 7) to obtain the accuracy, allowing for identification of slow deviations; the residual was noisy and the deviations were visible, but cluttered, making their automatic identification (see Section 3.4.5) work poorly. Therefore, we reviewed a series of various regression techniques—SVM, ANFIS-fuzzy models, lasso, ridge, regression trees, different variants of neural networks.

#### 3.3.3. Regression with Neural Network

We found the solution for the regression problem in neural networks [33], with their ability to solve the regression problems. However, we proposed some additional modifications besides classical feed-forward NN [34] tuning performed during NN engineering, such as the number of layers and neurons, and the selection of the training algorithm.

The classical feed-forward NN performed well for the regression of the position of a marker. The residuals should reveal distortions if the NN is not overtrained. However, fluctuations of the residual, which resemble pink noise, could cause false detections when thresholded. Therefore, we decided to mimic multi-start NN training, with *P*-fold replication of the target output $Y_{p}=\left[x,y,z\right]$ values. Thus, our prediction has the final step:

(10) $$\hat{Y}=\frac{1}{M}\sum_{m=1}^{M}{\hat{Y}}_{m}.$$

Random-valued initialization with the scaled conjugate gradient training method made each output replica follow the true values, and the errors (residuals) were not correlated, unless they represented actual distortion. Hence, their averaged residual values exhibited much lower noise levels, so it allowed us to reveal the slow artifacts with thresholding.

**Figure 8:**
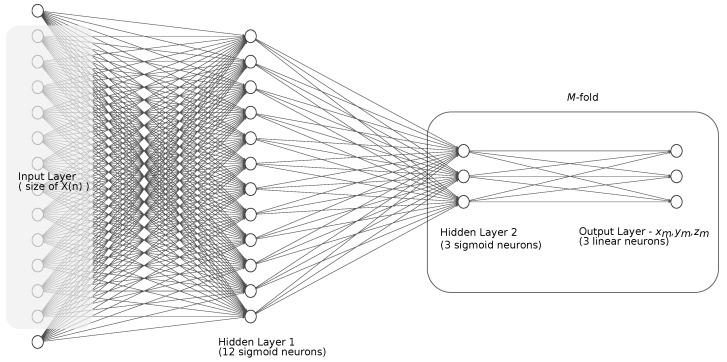
Architecture of the neural network used for regression.

The design of the NN structure is a kind of art, as there are no unambiguous rules or guidelines. Usually, it requires simulating with parameter sweeping for a domain of possible (feasible) numbers of layers and neurons, with a critical review of obtained performance (MSE or classification ratio) [35]. We shared that approach and reviewed the performance of NN using the test data. The NN architecture we employed is presented in Figure 8. It is ordinarily a fully connected FFNN, with two hidden layers—first containing 12 sigmoid neurons, second containing 4·M sigmoid neurons. The output is a three-valued x,y,z vector replicated *P* times—we used a five-fold replication. As input, we used a similar set of neighbor and parent coordinates to Equation (9); additionally, we enhanced it with the moving average of the own value of the marker. The latter could make the NN follow momentary slow changes; therefore, the window of a moving average (MA) should be notably larger than the lengths of the detected distortions (we assumed it to be 200 samples). By extensive testing, we also identified the number of previous values (=1) and the order of power used to raise the input data (L=2). Finally, each input vector *X* was long and assembled of certain parts, as given below:

(11) $$\begin{array}{cc} \; & \;X\left(n\right)=\left[\begin{array}{c} \;\overset{\text{current}\;\text{value}\;\text{and}\;k\;\text{former}\;\text{of}\;\text{parent}\;\text{marker}\;(p)}{\overset{︷}{x_{p}\left(n\right),y_{p}\left(n\right),z_{p}\left(n\right),x_{p}\left(n-1\right),\cdots ,z_{p}\left(n-k\right)}}\;,\;\;\;\;\\ \;\;\;\;\overset{\text{current}\;\text{value}\;\text{and}\;k\;\text{former}\;\text{of}\;\text{first}..\text{last}\;\text{siblings}}{\overset{︷}{x_{s1}\left(n\right),y_{s1}\left(n\right),z_{s1}\left(n\right),x_{s1}\left(n-1\right),\cdots ,z_{slast}\left(n-k\right)}},\\ \vdots \\ \;\;\;\;\;\;\underset{\text{current}\;\text{value}\;\text{and}\;k\;\text{former}\;\text{of}\;\text{first}..\text{last}\;\text{siblings}\;\text{raised}\;\text{to}\;L\text{th}\;\text{power}}{\underset{︸}{x_{s1}^{L}\left(n\right),y_{s1}^{L}\left(n\right),z_{s1}^{L}\left(n\right),x_{s1}^{L}\left(n-1\right),\cdots ,z_{slast}^{L}\left(n-k\right)}},\\ MA_{x}\left(n\right),MA_{y}\left(n\right),MA_{z}\left(n\right) \end{array}\right]. \end{array}$$

**Figure 9:**
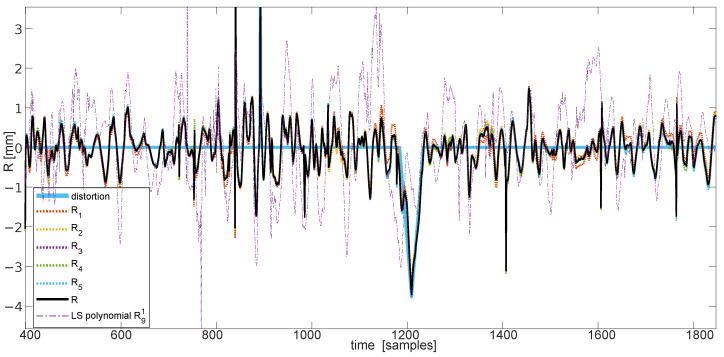
Actual distortion prediction and residuals—component ($R_{m}$) and final (*R*), compared with the polynomial residual $R_{9}^{1}$.

In Figure 9, we demonstrate the prediction results for the real sequence contaminated with artificially-introduced distortion. We can clearly observe that NN residuals contain the expected changes in signals, whereas residuals from the polynomial model are inconclusive.

### 3.4. Recognition and Classification of Distortions

**Figure 10:**
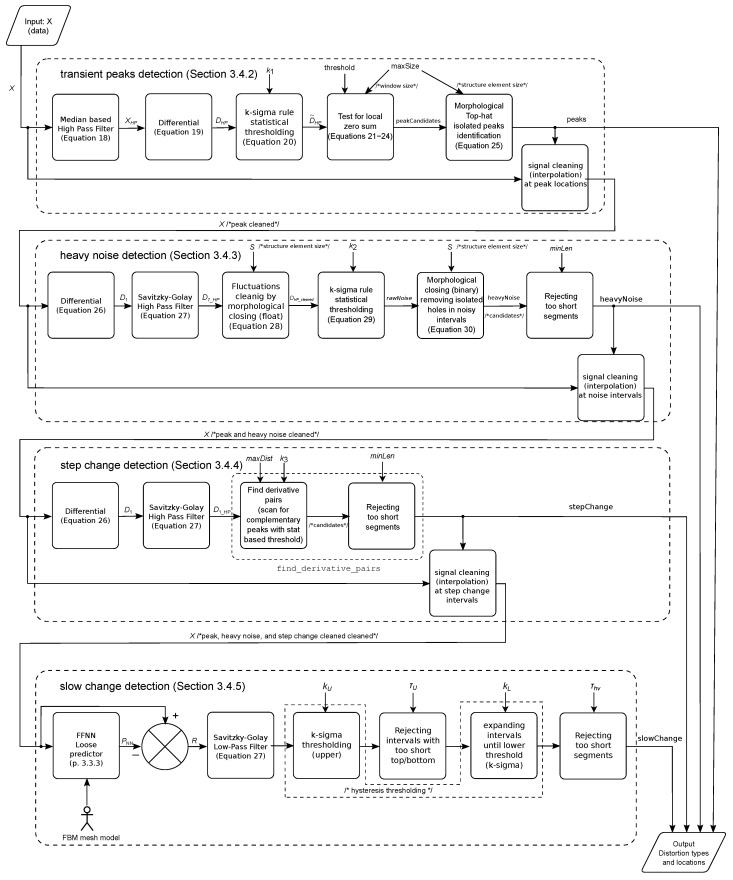
The detailed schematic of the proposed algorithm.

The detection process was organized into a strict pipeline consisting of four stages, where we detected distortions from the simplest to the hardest, along with the removal of the detected distortions (through interpolation after each stage). Such an approach ensures proper classification, otherwise we would have false positive classification due to the fact that subsequent methods could also be sensitive to simpler distortions, i.e., slow change would also be sensitive to rectangular distortion if the amplitude of distortion is sufficient. The detailed schematic of the dataflow in the algorithm is depicted in Figure 10.

The detection worked using the prediction residuals; therefore, we can see the deviations that cannot be explained by expected movements of markers described with the model. The choices of the models depended on the detected anomaly. For the sudden changes, these models were low-pass filters—median (for peaks) and Savitzky–Golay (for longer distortions); therefore, the respective high-pass filters acted as residuals. For the slow change, the model was FFNN and the residual was given explicitly as the difference between the signal and prediction.

To some extent, the residual values as deviations from a model can be considered innovations in the marker positions, resulting in position changes beyond the prediction. Therefore, minor residual values can be interpreted as normal motions, whereas large or sudden changes imply artifacts. Knowledge of the statistical properties of residuals allowed us to evaluate thresholds for detecting outliers of the normal variabilities of residuals.

#### 3.4.1. Locating Sudden Changes

Sudden changes are well detectable in the derivative of the basic signal, meanwhile slow changes require use of a base representation of residuals to measure the deviation. For the approximation of derivatives, we used differentials:

(12) $$\begin{array}{c} \Delta X(n)=X(n)-X(n-1). \end{array}$$

Discrimination of different types of sudden changes cannot solely rely on differentials. This is because a strong peak in ΔX notifies about the existence of a sudden change, but it does not bring information about the structure, i.e., the duration of the change or its neighborhood. Therefore, we employed mathematical morphology methods (MM) to analyze the shapes of those sudden changes. We used the following MM operations:

(13) $$\begin{array}{cc} \; & \text{Erosion}:E\left(n\right)=x\left(n\right)⊖S=\max(\forall_{j\in S_{n}}x\left(n-j\right)), \end{array}$$

(14) $$\begin{array}{cc} \; & \text{Dilation}:D\left(n\right)=x\left(n\right)⊕S=\min(\forall_{j\in S_{n}}x\left(n-j\right)), \end{array}$$

(15) $$\begin{array}{cc} \; & \text{Opening}:O(n)=x(n)\circ S=(x(n)⊖S)⊕S, \end{array}$$

(16) $$\begin{array}{cc} \; & \text{Closing}:C(n)=x(n)•S=(x(n)⊕S)⊖S, \end{array}$$

(17) $$\begin{array}{cc} \; & \text{Top-hat}:T_{w}\left(x\left(n\right)\right)=x\left(n\right)-\left(x\left(n\right)\circ S\right), \end{array}$$

where: x() is a 1D signal, *S* is the structuring element defining points to be taken into consideration (*j*), $S_{n}$ is the structuring element centered (translated) at *n*.

An additional morphological method involves seeking sudden changes; we implemented a scanning function (find_derivate_pairs(dX,T,maxlen)), which looked for opponent differential pairs dX, exceeded the threshold level T, and was no more distant than some presumed maximal length maxlen. It results=ed in binary decision variables marking located ranges. The function parameters—threshold and distance—depend on the data characteristics and sampling frequency.

#### 3.4.2. Identifying Single Peaks

It is the first stage of processing. Single peaks and heavy noises are two appearances of the same short-term distortion—the key difference is that single peaks are isolated within some neighborhoods, whereas in heavy noise segments, numerous peaks occur next to each other. To identify the isolated peaks, we employed the following sequence of operations.

First, we removed low frequencies (presumed to be legitimate) using a median filter:

(18) $$X_{HP}=X-\mathrm{median}\left(X,window\right),$$

where the window size should be several times larger than the maximal peak length.

Then, we calculate differential:

(19) $$D_{HP}\left(n\right)=X_{HP}\left(n\right)-X_{HP}\left(n-1\right),$$

which is cleaned of non-interesting low values using thresholding:

(20) $${\tilde{D}}_{HP}\left(n\right)=\left\{\begin{array}{cc} D_{HP}\left(n\right), & \mathrm{if}\;D_{HP}\left(n\right)>threshold_{1}\\ 0, & \mathrm{otherwise} \end{array}\right\}$$

Next, the identification of probable peaks is based on the assumption that the differential sum is local to small values (in theory ≈ 0), whereas the local sum of absolute values of the differential is high. Thus, we calculated these sums within window $W_{n}$:

(21) $$movSum\left(n\right)=\sum_{j\in W_{n}}{\tilde{D}}_{HP}\left(n-j\right)$$

and

(22) $$movSumAbs\left(n\right)=\sum_{j\in W_{n}}\left|{\tilde{D}}_{HP}\left(n-j\right)\right|$$

which are then tested as:

(23) $$ampRatio\left(n\right)=\frac{movSumAbs(n)}{\left|movSum(n)\right|}$$

when the ampRatio is larger than the assumed threshold (we assumed 5), it implies there is a peak candidate:

(24) $$peakCandidates\left(n\right)=\left\{\begin{array}{cc} 1, & \mathrm{if}\;ampRatio\left(n\right)>threshold_{2}\\ 0, & \mathrm{otherwise} \end{array}\right\}$$

Finally, to identify single peaks only, we employed binary top-hat, which rejects peaks within the neighborhood defined by the structuring element. It allowed us to keep isolated peaks only:

(25) $$peaks=T_{w}\left(peakCandidates,S\right)$$

Tunable parameters of the stage are:

- window—for the moving average, we assumed it to be 19 samples long;
- $threshold_{1}$—it is calculated statistically from the data using $k_{1}\cdot \sigma$ of $D_{HP}$—w employed 3·σ as a default value; however, any $k_{1}$ can be provided as the parameter;
- $threshold_{2}$—(anti-sensitivity) for the ampRatio;
- maxSize—(default 5), which declares the maximal size of the expected peaks; it affects the size of moving sum windows $W_{n}$, which is 2·maxSize+3 samples long, it also defines the size of the linear structuring element for morphological operations *S*.

#### 3.4.3. Heavy Noise

Heavy noise detection is somewhat similar in design to isolated peak detection, but there are differences in the details. Foremost, we assume that the input data are already clear of isolated peaks. First, we calculate the differential:

(26) $$D_{1}=X\left(n\right)-X\left(n-1\right),$$

from which we remove low frequencies using a high pass variant of the Savitzky–Golay filter (Equation (7)):

(27) $$D_{1\_HP}=SavitzkyGolayHiPass\left(D_{1},L,M\right).$$

Next, we remove small fluctuations within the prospective areas of high values in $D_{h}p$ with morphological closing (float):

(28) $$D_{1\_HP\_cleaned}=\left|D_{HP}\right|•S.$$

These values are now thresholded:

(29) $$rawNoise\left(n\right)=\left\{\begin{array}{cc} 1, & \mathrm{if}\;D_{1\_HP\_cleaned}>threshold\\ 0, & \mathrm{otherwise} \end{array}\right\}.$$

Raw noise intervals are finally cleaned by removing holes using morphological closing, but the binary variant this time:

(30) heavyNoise=rawNoise•S.

Finally, heavy noise segments shorter than the minLen attribute are rejected.

Tunable parameters of the heavy noise detection stage are:

- threshold—this is calculated statistically from the data using $k_{2}\cdot \sigma$ of $D_{HP}$—w employed 2·σ as a default value; however, any *k* can be provided as parameter;
- Minimal length of the segment (minLen), which is used to define the linear structuring element *S* as 2·minLen−1; we assumed minLen=20 samples;
- Default parameters of Savitzky–Golay are *L* = 5, *M* = 13.

#### 3.4.4. Step Change

Step change is another differential-based detection; it resembles the two former detections. It requires removing isolated peaks and heavy noise areas. After that, identification of rectangular-like changes becomes a simple problem.

**Figure 11:**
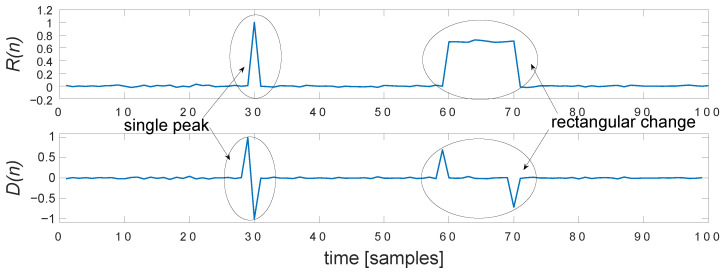
Appearance of sudden distortions in residual/high-pass (R(n))—single peak and step change, and their corresponding peak pairs in differential D(n).

The first two steps are shared with heavy noise detection. We compute $D_{1}$—differential (Equation (26)), which is then high-pass filtered with the Savitzky–Golay filter (Equation (27)), so we have $D_{HP}$. Next, we employ simple scanning with find_derivate_pairs, which seeks the areas between the complementary pairs of differential peaks (above threshold); we interpret it as a rectangular distortion, as shown in Figure 11. This scanning requires setting up two parameters—minLen and maxDist, identifying the minimal length of a step change, and maximal distance of searching.

Tunable parameters for the step change detection stage are:

- threshold—this is calculated statistically from $D_{HP}$ using $k_{3}\cdot \sigma$ of—we employed 3·σ as a default value; however, any $k_{3}$ can be provided as parameter,
- Minimal length of the segment (minLen); we assumed minLen=20 samples;
- Maximal searching distance maxDist; we assumed 200 samples as the default value.
- Default parameters of Savitzky–Golay are the same as for heavy noise *L* = 5, *M* = 13.

#### 3.4.5. Identifying Slow Changes

**Figure 12:**
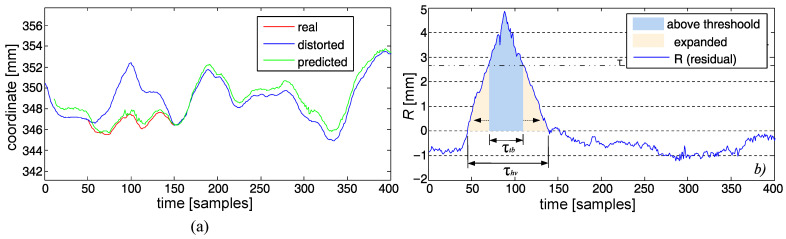
Slow detection: (**a**) original, predicted, and distorted signal, (**b**) residual with hysteresis thresholding.

The slow changes of the positions (Figure 12a) involve a class of distortions notably different from the other ones, requiring a separate approach for detection due to the fact that its nature makes it hard to detect with differential analysis. We employed a loose NN model predictor ($P_{NN}$) as described in Section 3.3.3. It predicts the proper marker position on the basis of its neighbor markers (parent and sibling). The deviations from such a model (*R*—residuals) were analyzed, looking for notably large and long deviations, identified as artifact hills or valleys.

(31) $$R\left(n\right)=X\left(n\right)-P_{NN}\left(n\right)$$

The hills and valleys could be of a different scale; the length and ’differential’ can vary significantly. Moreover, predictor fluctuations at the turning points can also seem similar to short-term slow changes (of small amplitudes) appearing when the predictor cannot follow the change of value. Though, based on the statistical properties of the residual of the prediction and on the fact that we know that the distortion should be rather long (as it is an accumulated reconstruction error), one can make certain assumptions allowing for detection of the distortions.

We used ‘scanning of values’ of the Savitzky–Golay smoothed residual of regression with hysteresis thresholding. It can be described in the following steps (see also Figure 12b):

1. Smoothed the *R* with the Savitzky–Golay low-pass filter (*L* = 7, *M* = 11—parameters heuristically tuned).
2. The upper threshold $T_{u}$ was set up with a $k_{U}\cdot \sigma$ rule of a thumb—in our case, three times $\sigma_{R}$ was selected ($k_{U}=3$) as the default would identify the significant tops and bottoms of the hills and valleys.
3. If the top or bottom lengths were shorter than some minimal $\tau_{U}$, we skipped it (0.2 s—20 frames in our case), assuming it to be short-term fluctuation.
4. After the identification of a top/bottom value, we looked for the rest of a distortion (below threshold)—the marked range expanded both sides iteratively (in the past and future) until the residual value went below/above the lower threshold $T_{L}$, obtained with $k_{hv}\cdot \sigma_{R}$ with $k_{hv}=0.5$ as the default value.
5. If the overall located distortion (hill/valley) was shorter than some value $\tau_{hv}$ (50 frames—0.5 s), it was omitted, as one can consider it a short-term fluctuation of the predictor.

## 4. Verification of the Method

The verification of the efficiency of the proposed approach was three-fold. In the first experiment, we analyzed the efficiency of the distortion classification using synthetically generated distortions in artifact-free sequences, which allowed us to provide some statistics on the classification efficiency. In the second test, we compared the performance of the proposed approach to the human operators of various experiences—from novice to experts. The last test was connected with the applicability of the artifact classification for the data cleaning with a pool of generic reconstruction algorithms.

### 4.1. Materials and Methods

#### 4.1.1. The Data

For testing purposes, we used data sets acquired for professional applications in the motion capture laboratory. The sequences were obtained at the PJAIT human motion laboratory using the industrial-grade Vicon MX system. The system capture volume was 9 × 5 × 3 m. To minimize the impact of external interference, such as infrared interference from sunlight or vibrations, all windows were permanently darkened and cameras were mounted on scaffolding instead of tripods. The system was equipped with 30 NIR cameras manufactured by Vicon—10 pieces of each kind: MX-T40, Bonita10, Vantage V5.

During the recording, we employed two system configurations—a standard animation pipeline, where data were obtained with Vicon Blade software (using a 53-marker setup) and a typical biomechanical setup using Vicon Nexus software (using a 39-marker setup). The trajectories were acquired at 100 Hz; by default, they were processed in a standard, industrial quality way, which included manual data reviewing, cleaning, and denoising, so they could be considered distortion-free. However, depending on the experiment, different variants of the recordings were used in experiments; these were raw unprocessed data, processed (cleaned), and artificially-modified variants with controlled amounts and locations of distortions. Information, on which variant was used is provided in the detailed description of the experimental protocols.

The two recordings used in the experiment illustrate the ability to adapt to the different marker settings used in different application areas. The first sequence was clean with no errors, but relatively comprehensive—all the limbs were moved and the feet freely swung in random directions; therefore, it could be challenging for the predictor. The second sequence contains quite a lot of reconstruction errors in its raw form, so we had material to compare the results to the human operators.

#### 4.1.2. Experimental Protocols

We planned the first experiment (E1) to test the performance of the method proposed in Section 3, using default parameters for a controlled dataset, with a perfectly clean sequence and controlled artificial distortions. It involved the first recording from the Table 1—‘Static’, which was manually cleaned by an expert, so it was artifact-free ground truth. Next, we introduced distortions at random locations (randomly drawn markers) and random amplitudes—see noise contamination procedures given further in Section 4.1.3.

Companion results were additionally acquired in the experiment to compare the results obtained in E1 to the existing methods of anomaly detection. Since artifact classification is a completely new approach, it has been a bit difficult to select suitable methods to compare with. We refer to the two generic methods [36] for anomaly detection in the time series: three-Sigma move (M3S), which employs mean and variance moving; the other one is the Hampel filter (HF), which is based on the moving median and median absolute deviation (MAD), which are more robust measures.

During the experiments, we kept track of the distortions and their types; therefore, we were able to verify whether the error classification was correct. The criteria for evaluation in the artifact recognition task are pretty straightforward—classification rates (true and false recognition) presented as a confusion matrix. The simulations were performed for three distortion shares 5%, 10%, and 20%; each was executed 1000 times and the results are aggregated as average confusion matrices (rounded). For each class, we calculated the following measures:

(32) $$\begin{array}{cc} \mathrm{sensitivity}\;(\mathrm{true}\;\mathrm{positive}\;\mathrm{rate}): & \;TPR=\frac{\mathrm{tp}}{\mathrm{tp}+\mathrm{fn}}\cdot 100\%, \end{array}$$

(33) $$\begin{array}{cc} \mathrm{miss-rate}\;(\mathrm{false}\;\mathrm{negative}\;\mathrm{rate}): & \;FNR=\frac{\mathrm{fn}}{\mathrm{tp}+\mathrm{fn}}\cdot 100\%, \end{array}$$

(34) $$\begin{array}{cc} \mathrm{fall-out}\;(\mathrm{false}\;\mathrm{positive}\;\mathrm{rate}): & \;FPR=\frac{\mathrm{fp}}{\mathrm{tp}+\mathrm{fp}}\cdot 100\%, \end{array}$$

(35) $$\begin{array}{cc} \mathrm{precision}\;(\mathrm{positive}\;\mathrm{predictive}\;\mathrm{value}): & \;PPV=\frac{\mathrm{tp}}{\mathrm{tp}+\mathrm{fp}}\cdot 100\%, \end{array}$$

Additionally, two more measures were employed to quantify performance. The F-score is a scalar describing the efficiency of overall classification for all classes [37]—its values are between 0 (no proper classification) and 1 (perfect classification). From various equivalent formulas, we chose the following one, because it was simple to adapt to the multiclass problem:

(36) $$F=\frac{\mathrm{tp}}{\mathrm{tp}+\frac{1}{2}\left(\mathrm{fp}+\mathrm{fn}\right)}.$$

The other measure was Matthews correlation coefficient (MCC) [38], which is a quality measure intended for characterizing the classification efficiency for imbalanced populations of classes (as in our cases). Its values scale between −1 for no classification and 1 for perfect classification. The formula is given as:

(37) $$MCC=\frac{\mathrm{tp}\cdot \mathrm{tn}-\mathrm{fp}\cdot \mathrm{fn}}{\sqrt{\left(\mathrm{tp}+\mathrm{fp})(\mathrm{tp}+\mathrm{fn})(\mathrm{tn}+\mathrm{fp})(\mathrm{tn}+\mathrm{fn}\right)}},$$

where cardinality of classifications are denoted as: tp—true positive classifications, fp—false positive, tn—true negative, fn—false negative.

The second experiment (E2) involved comparing the performance of the proposed method using default parameters to four operators of the mocap facility, with different levels of experience—two beginners (2 and 3 months of experience), one intermediate (1.5 years experience), and one expert (10 years of experience). The number of respondents was small and imbalanced because it was hard to find volunteers of that expertise. Additionally, we preferred to control and monitor the reconstruction process—including the software version and its setting—so we needed them in our lab facility. The results we present here represent all the people who worked in the lab and who agreed to do some work for us. Since a mocap operator is a rare profession, every response is informative; therefore, we preferred to present the imbalanced number of operators than to remove one of the respondents.

The test is intended to be a qualitative verification of the proposed approach and to verify the proposal using real life data. We used the raw form (not cleaned) of the ‘Sitting’ recording, which contained all kinds of distortions. Such data were used against the proposed detection algorithm. Apart from automatic processing, the four operators conducted normal data screening and cleaning. These manual processing steps, using Vicon Nexus software, were a standard approach in the lab, which is in everyday usage in the facility. In the final step, the results obtained by the algorithm and four operators were reviewed by an expert—a human mocap system operator with long experience in data editing and cleaning. The results are reported as raw numbers of distortions located, compared, and verified against human judgment.

The last experiment (E3) involved verification of the applicability of the proposed approach. It was intended to be a proof-of-concept of the targeted distortion cleaning. Therefore, it used different variants of static person sequences with the distortions of variable intensities and durations introduced into the recording—taking 5%, 10%, and 20% of the overall length—similar to E1. In the tests, we employed our algorithm with the default settings as a detector, which then was combined with the following reconstruction methods: Savitzky–Golay (13th order polynomial over 101 samples window), linear interpolation, spline interpolation, and FFNN prediction (as given in Section 3.3). Each method was applied in the locations of the detected artifacts only, the rest of the signal remained intact. All distortions were simulated separately in this case, with a randomized location (marker), time, duration, and an amplitude with 10 mm of average value and 4 mm of std deviation. The simulations of contamination–detection–reconstruction were performed 200 times; for each fold we obtained a quality measure, which was finally averaged. We assumed the root-mean-square error (RMSE) as the measure of quality; it was computed over all the coordinates and samples in the considered sequence:

(38) $$RMSE=\sqrt{\frac{1}{K\cdot N}\sum_{k=1}^{K}\sum_{n=1}^{N}{\left({\hat{x}}_{k}\left(n\right)-x_{k}\left(n\right)\right)}^{2}}\;,$$

where: *K* is a number of variables in the time series, *N* is the number of samples, x() is the original value, $\hat{x}\left(\right)$ is the reconstructed value.

#### 4.1.3. Artifact Contamination Procedure

The procedure of distortion contamination, which was employed in E1 and E3, introduced artifact distortions into the sequences in a controlled way—we logged the type, duration, location, and amplitude of the distortion. The contamination could include mixtures of all kinds in equal proportions. The key parameter characterizing the experiment was time share (distorted time fraction), for which distortions were generated. For the interpretation clarity, we ensured that only one distortion at a time occurred; therefore, the time share denotes that a distortion occurs at a given fraction of time. The sequence of distorting the signal is as follows: first, we drew locations to contaminate with ’bulk’ distortions—slow, step changes, and heavy noise; next, we seeded randomly isolated peaks. Distortion parameters were set up randomly for each instance of distortions:

- The sign was a +1/−1 value drawn with equal probabilities;
- The amplitude was a Gaussian random variable with assumed amplitude and standard deviation (in the tested cases: μ=5or10 mm and σ=0.4·μ); these values were used to scale the peak of the rectangle or triangle distortion and as the standard deviations in the heavy noise area;
- Distortion durations and intervals were part of the Poisson process; an average length of distortion was set up to 50 samples, and the interval length was adjusted according to the duration of the sequence and the target amount of the given distortion.

The distortions introduced were quite demanding for the detection procedure. The amplitudes were on average small (5 and 10 mm) and of short (0.5 s) duration; therefore, we could assume that the synthetic tests were rather rigorous and more difficult to detect than in real life scenarios.

### 4.2. Results and Discussion

The outcomes of all three experiments are provided in the successive sub-paragraphs. They are accompanied by interpretations and discussions.

#### 4.2.1. Synthetic Distortion Classification

**Figure 13:**
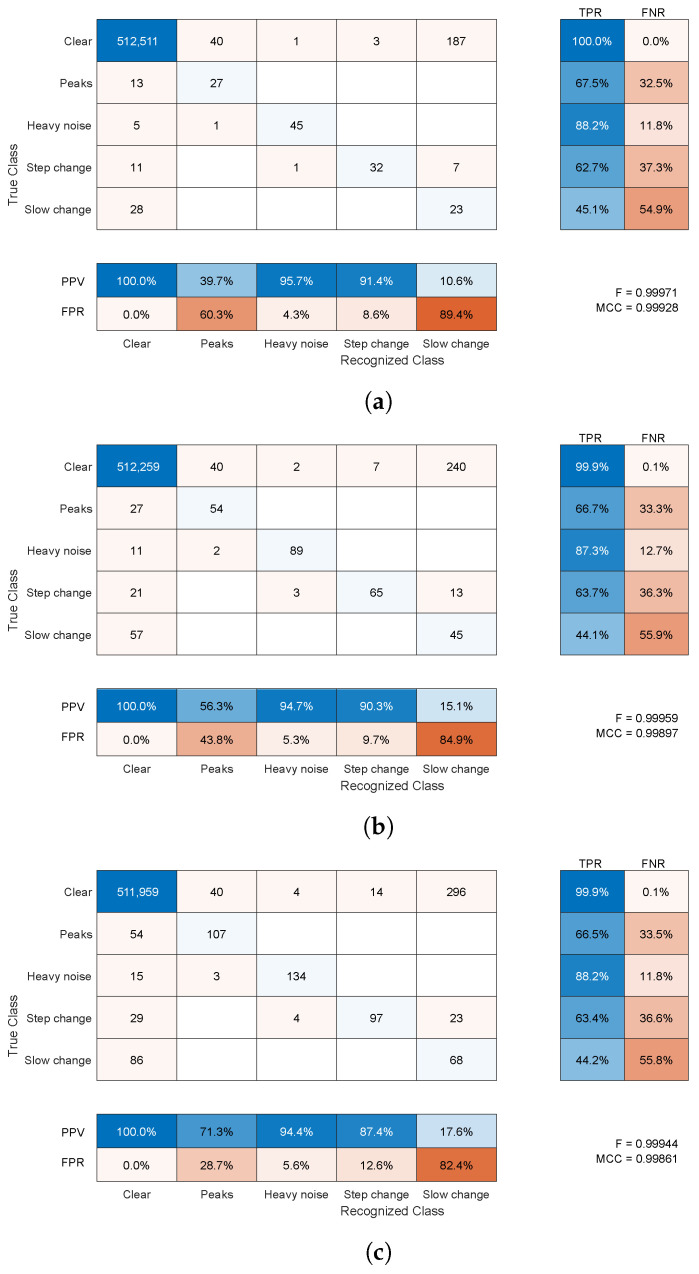
Average confusion matrix for detection of synthetic noises for a 1000-fold simulation with 5-mm average amplitudes of distortions and shares: (**a**) 5%, (**b**) 10%, (**c**) 20% of time (blue—successful results, red—faulty).

**Figure 14:**
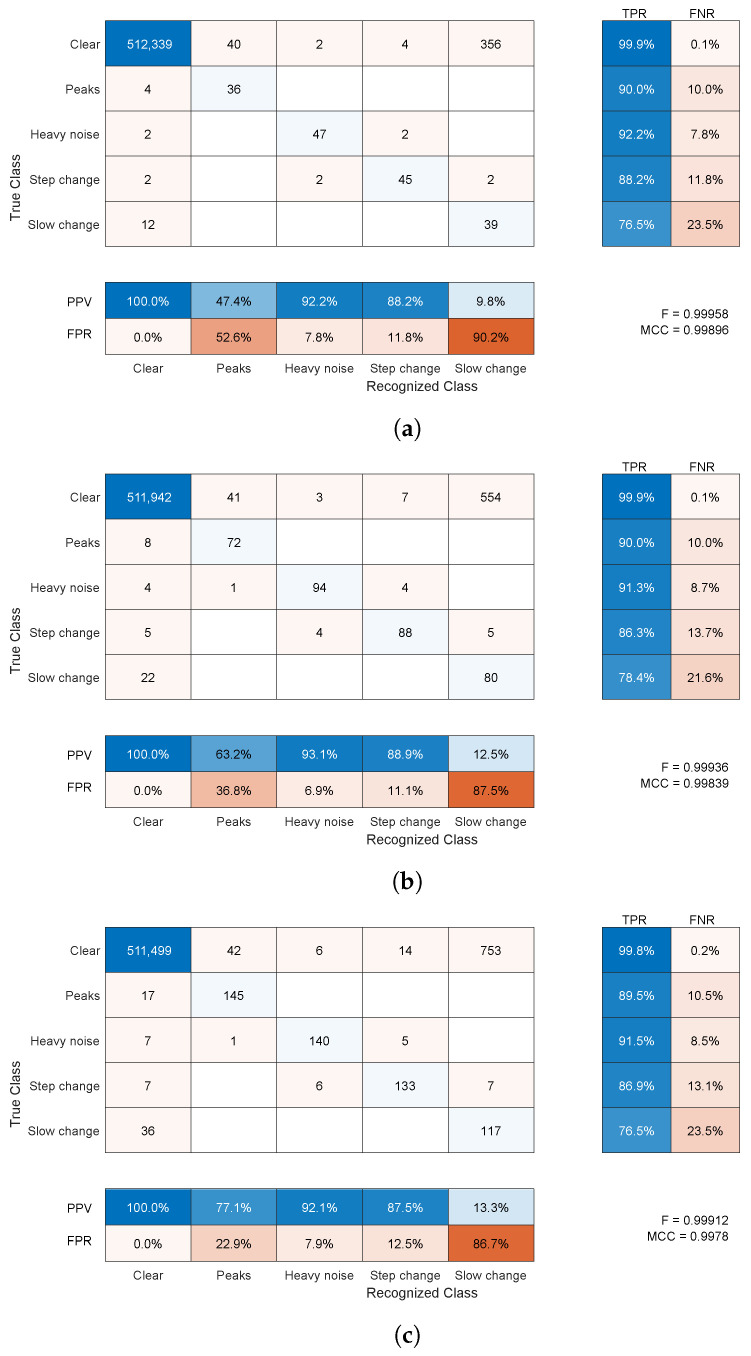
Average confusion matrix for detection of synthetic noises for a 1000-fold simulation with 10-mm average amplitudes of distortions and shares: (**a**) 5%, (**b**) 10%, (**c**) 20% of time (blue—successful results, red—faulty).

The classification for synthetic noise outcomes are demonstrated in Figure 13 and Figure 14 for average amplitudes of 5 and 10 mm, respectively. The raw results in confusion matrices demonstrate the average number of samples (rounded toward the whole sample) assigned to specific classes (correct or not) for 1000 simulations. They present averaged confusion matrices for three distortion shares 5%, 10%, and 20% of the overall time of the sequences with two average amplitudes—5 and 10 mm. According to the length of the recording in a simulation, the contamination procedure should produce approximately 160, 320, and 640 distorted samples, respectively, of each distortion type, and should be in equal proportions.

**Figure 15:**
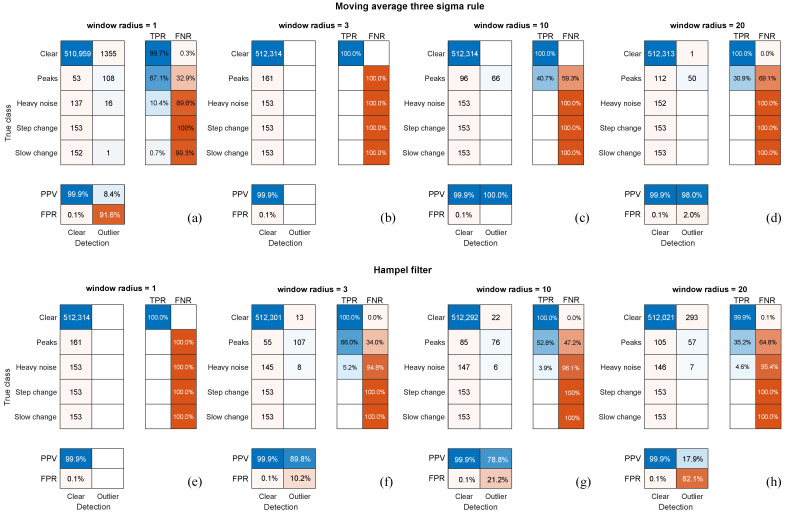
Averaged confusion matrix for detection with M3S (**a**–**d**) and the Hampel filer (**e**–**h**) of synthetic anomalies for a 1000-fold simulation with 10-mm average amplitudes of distortions and a share 20% of the time (blue—successful results, red—faulty).

The comparative results for the generic anomaly detectors, HF and M3S in different configurations of the moving window, are demonstrated in Figure 15 as confusion matrices. The true anomalies are subdivided into classes, whereas the output is binary, whether it is detected as an outlier or not. The figure presents only the best of the results for 10 mm of amplitude and a 20% share of distortions, so it should be compared with the results in Figure 14c. We did not include the results for other distortion configurations, because they were either very similar (about recognition ratios) for 10 mm amplitudes or significantly worse for 5 mm.

Regardless of the number of distortions (shares), the results were pretty consistent; they were also very similar for numerous additional simulation runs, which are not included here. The fractions of true and false classifications hold across the runs. The same almost holds for F-scores and MCC values to a lesser extent. Therefore, the confusion matrix is the most informative presentation of results as it is near 0.999 for both amplitudes and all distortion shares—these large F-score/MCC values are due to the dominance of the properly classified clear signal samples. Nevertheless, they offer some insight into the results, with an increase in the share of distortions in the test sequence, we observe a very slight decrease in the classification performance expressed with the F-score/MCCs. These differences stem mainly from a slightly increased number of clear samples falsely classified as artifact-contaminated, since the classification rates remain on par between the artifact shares.

Each specific class requires separate insight into the results. These are as follows:

- The clear signal was identified properly for more than 99% of samples; a negligibly small amount of distorted samples was erroneously identified as clean signals (compared to the overall cardinality of the class).
- For the peak change, sensitivity was approximately 66% and 90%, and the main misclassification was in a clear signal; this class was not a cause of confusion for the other classes compared to a clean signal (usually below FPR=50%).
- Heavy noise sensitivity was above 88%; the main confusions were step change and a clear signal; this class was rarely erroneously recognized in place of the others (FPR = 4–8%); the main confused class was a clear signal.
- For the step change, sensitivity was approximately 70% and the main confusion was slow change; this class was erroneously recognized in place of others at a moderate rate (FPR = 12–27%)—here, a clean signal and heavy noise were wrongly identified.
- For the slow change, TPR was a bit more than 50% and the main confusion was a clear signal; this class was often difficult and erroneously recognized in place of others (FPR = 80–90%)—usually, it was a clear signal, but a step change and heavy noise were also wrongly identified.

Considering the above results, the proposed method has moderate to high sensitivity (depending on the class) and quite high precision for all classes but one (slow change). False negative detection (having relatively small values) was way more undesirable than the others; this was notable from a practical point of view. False positive, or detecting a wrong class of the distortion, would still result in pointing out the operator to the potential error location, or in the case of automatic error filtering, it would lead to the use of a repair procedure (see Section 4.2.3).

The difficulty in identification of slow change was expected; it comes from the fact that this change can be subtle and poorly distinguishable in predictor residuals, which resemble pink noise in our case. The latter is also a cause of high FPR. We analyzed alternative regression methods as a model—neural networks (simple FFNN and NARX-NN models), ridge, lasso, and SVR. However, the results were either poor or impractical (due to long training time), or both. On the other hand, a false positive error (quite frequent) is of much lesser importance than a false negative; the former might result in suggesting additional locations to the operator for reviewing, or using the interpolating method, which should not degrade the signal significantly; whereas the latter might result in preserving the distortion in the signal.

The comparison to the other anomaly detectors shows that the proposed solution outperforms these methods. In the best-tested configurations, they were capable to identify (as outliers) approximately 2/3 of peaks, and a small part (5–10%) of heavy noise-contaminated samples (usually the initial ones before the local variance or MAD value increased in the presence of the noise, so much that the thresholds did not intercept the noise anymore). The two other signal anomalies—step and slow change—remained invisible to these methods (excluding a single accidental case in M3S. Meanwhile, our approach (Figure 14c) was capable of identifying approximately 90% of the first two anomalies and above 75% of the latter two, which were ‘invisible’ to the generic detectors.

Regarding false detections, in most cases (all but one), both the generic methods returned small numbers of false positive detections. We attributed this to the overall low sensitivity of these methods, and in cases when the detection rates were a bit higher, false positives were also elevated (Figure 15a,f,g).

Both the M3S and Hampel filter are methods that consider each coordinate separately; therefore, they do not use inter-marker dependencies. This allowed us to identify individual anomalous coordinates. Regrettably, because of the latter, we had to reject the other (more sophisticated) methods of anomaly detection based on machine learning, such as clustering, one-class SVM, or autoencoders as insufficient. In their basic variants, they considered the whole frame of a recording as a single observation with coordinates as features. This implies that they could potentially point out anomalous frames in sequence, but they would not identify what marker/dimension is the problem. One might think of adopting such approaches to anomaly detections; however, it would require a separate in-depth study.

#### 4.2.2. Comparing to Human Operators

Table 2 comprises the numbers of detected distortions in the recording processed in a standard pipeline, and the recording processed by each of the four operators. The detected distortions were compared and verified by human operators, who either approved the classifications or rejected them.

**Figure 16:**
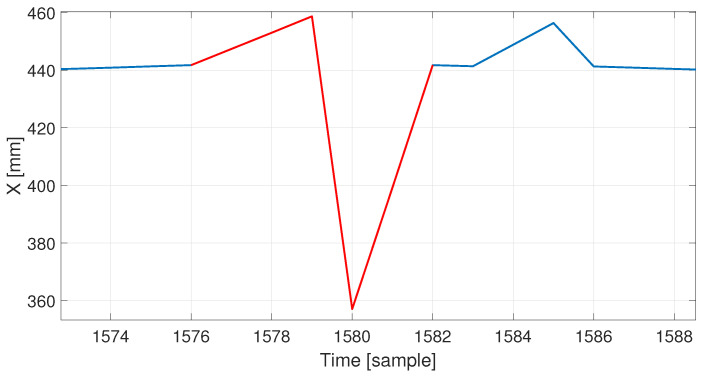
Additional combined distortion types (red line indicates faulty values).

In the recording processed by the machine, the proposed algorithm found 29 errors—peaks, step, and slow changes; 16 of these errors were correctly classified. The algorithm classified sudden, very dynamic hand movements in a relatively static recording as slow changes (13 incorrectly classified errors). In addition, the algorithm did not detect four errors; after careful analysis, it turned out that these errors did not belong to any of the previously defined classes—they were a combination of a single peak and a slow change. An example of such an error is shown in Figure 16. Such an artifact (having a relatively large amplitude) could be intercepted if the slow change detector had different parameters, but it would require setting up a smaller value for a minimal length of an artifact. It could result in an increased number of false positives for this class, as this parameter prevents false alerts of the short-term fluctuations of the NN predictor. It is worth mentioning that the slow change detector reacts properly for a mixed class of artifacts—step changes followed by slow relaxing to the proper trajectory (or slow error accumulation followed by immediate correction) do not cause detection for any of the detectors based on finding derivative pairs, but they properly trigger detection for the deviations from the predictor if they are long enough. In fact, all the deviations that are large and long enough would be identified as slow changes. It is a matter of human interpretation whether we can name them as slow; however, that name distinguishes them well from all of a sudden changes we identified based on the differential analysis.

In the recording processed by the expert, the algorithm again incorrectly classified the hand movements as slow changes. The result was similar for the intermediate operator, with the difference that the algorithm found two errors omitted by the human (two small peaks).

In the case of a recording repaired by beginners, both the algorithm and the expert found more errors after the repair than before. This was due to the selection of an inappropriate method of repairing a given artifact. For example, when the distortion occurred only on one axis, the person, in order to correct the error, removed the marker trajectory for those few frames when the error occurred. This resulted in the creation of an additional gap, which the beginner operator filled using simple interpolation. In the case of longer artificial gaps, interpolation caused the data to be incorrectly reconstructed, and the errors no longer appeared on one axis, but on all three.

#### 4.2.3. Applicability Testing

**Figure 17:**
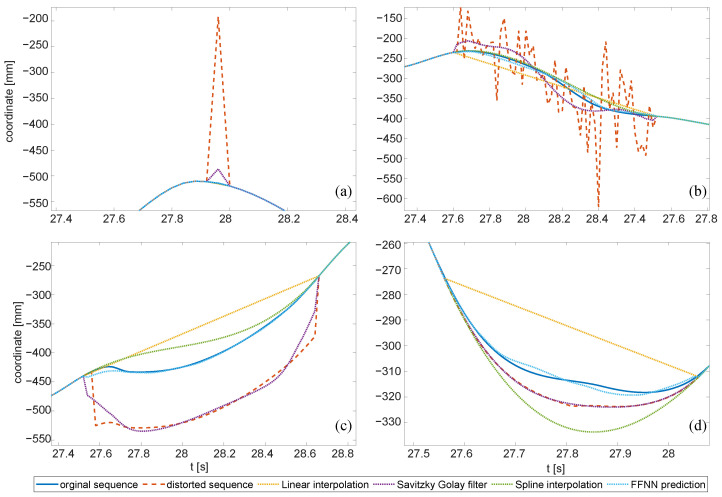
Artifacts and the removal with methods tested in E3: (**a**) single peaks, (**b**) heavy noise, (**c**) step change, (**d**) slow change. Please mind the various scales in the axes.

The results are presented in Table 3, Table 4 and Table 5. Each field presents the averaged *RMSE* of a 200-fold repeated simulation process, distorting the test sequence and its reconstruction using various procedures. Each distortion type was considered separately. It allowed us to quantify how each reconstruction method reduced the distortion. Figure 17 illustrates the reconstruction results, demonstrating the ground truth, distorted signal value, and outcomes of four variants of reconstruction. In the tables, for each distortion type and reconstruction method, we compare two *RMSE* values: hypothetical perfect classification and actual classification with the proposed algorithm.

In the results, we recognize the different efficiencies of the tested reconstruction methods for different distortions. We could also clearly observe that the efficiency of detection of the artifacts directly affects the ability to restore the signal. The key observations are summarized in a few points:

- Peak changes were effectively removed with interpolation methods—simple linear or spline (piecewise cubic polynomial); the other two methods in perfect detection would not offer even comparable efficiency, yet in actual classification, they offered just slightly worse performances.
- FFNN offered the best performance for all ‘bulky’ distortions (of longer durations), both hypothetical and classified cases.
- Heavy noise, aside from FFNN, was well cleaned with the Savitzky–Golay filter (see Figure 17b).
- Step changes could be effectively removed with FFNN only.
- Slow changes were the most contradictory—the only appropriate reconstruction method was FFNN; in the case of perfect detection, the efficiency was high, but due to the limited actual detection, the results were quite poor. These results correspond well to the detection of slow changes in E1—low sensitivity and high fall out.

The above outcomes of reconstruction are just preliminary results. They should be further analyzed in a separate study for the possible reconstruction methods involving other predictors, interpolations, the rigid body model, and projections on geometric constraints.

### 4.3. Results Recap

All above outcomes indicate that the proposed approach offers reasonable results. Experiment E1 proves that sudden changes are way easier to detect than slow changes, but slowly accumulated errors are also detectable (with less efficiency). It is mainly a matter of predictor efficiency, so the amplitude of distortions is a key factor affecting artifact locating.

Another disputable aspect is the fact that there is no simple possibility to compare the detection and classification efficiencies to the other solutions, as this work is the first proposal in this area. The only method that is somewhat comparable to ours is publicly unavailable. Moreover, it is capable of segmenting artifacts only and cannot classify distortions, so the performance comparison, if possible, would be very limited.

Comparing the reconstruction efficiency (as in E3) to the existing repairing method—the classification efficiency would not only be evaluated, but also (foremost) the quality of the reconstruction algorithm.

However, we were able to compare the efficiency of our solution to the industrial-grade software in an indirect way, as described in E2. Automatic repairing algorithms offered within modern software (Vicon Nexus) could degrade the sequence (increase the number of artifacts)—this is well illustrated by the results of the novice operators, who used automatic repairing, resulting in an increased number of artifacts.

Additionally, referring to our experience with the state-of-the-art software, when using the ‘find bad data’ function in the Vicon Blade software, it is not the best option to let the software do everything for us. This method requires three parameters: threshold (allowed deviation in millimeters), cut-off (the cut-off frequency), and sensitivity (amplifies the effect of the cut-off frequency). Unfortunately, this method requires one to set different values to find different errors. Moreover, these parameters may be different for each recording, e.g., more dynamic recordings require increasing the threshold. Another drawback is the fact that each marker trajectory is treated separately, with no inter-marker relations, so slow changes are invisible to that method.

Finally, in the proposal, we presumed that (for certain markers) there was one distortion type at a time. This might not be true in some cases; however, this matters in rare cases, since one fault usually happens at a time, or one is dominant and clutters the others. Some combinations of errors can be well distinguished (e.g., peaks/noise during slow change), whereas others cannot (step changes within noise). Therefore, as we demonstrated in E2, such an algorithm cannot replace experienced operators but can be of assistance, making the jobs faster and less burdensome.

## 5. Summary

In this article, we addressed the issue of artifacts occurring in the mocap signal. We proposed a method for their detection and demonstrated how to employ the detection method to improve signal fidelity. The method proposed in this article seems to be quite effective for sudden changes, and it can detect distortions of relatively small amplitudes. As for the slow changes, their outcomes are moderate, since we observed a relatively large number of false positive detections. However, we expected that this class of distortions might be difficult to detect. This topic is worthy of further study.

Compared to human operators, the proposed solution cannot outperform experienced professionals; however, it offers a notably better performance than a novice operator. On the other hand, even for an expert, it can save time by suggesting locations to review.

The proposal could be adopted in existing software as an optional step of signal refinement and/or for automatic support for the mocap sequence editors. Further improvements are still possible, but require additional research, such as employing better predictive models. Moreover, the engineering approach could be beneficial for detection efficiency. One improvement could be to detect distortions for all three coordinates of a marker, jointly, since distortions usually occur in more than a single coordinate. Moreover, studying the reconstruction methods is a topic that we plan to investigate in the future.

## Figures and Tables

**Table 1:** List of mocap sequence scenarios used for the testing.

No.	Name	Scenario	Duration	Difficulty
1	Static	Actor stands in the T-pose in the middle of the scene, looks around, and shifts from one foot to another	22 s	easy, static
2	Sitting	Actor stands in the middle of the scene and then sits on a chair; actor stands again after a few seconds and repeats this three times	29 s	occlusions

**Table 2:** Comparing the number of distortions located by the proposed method to the human operator (E2).

Operator	Seq. No	Recording	Errors Identified by		Error Verification		
			Human	Algorithm	Approved	Rejected	Missed
None	2	Sitting	—	29	16	13	4
Expert	2	Sitting	20	9	0	0	0
Intermediate	2	Sitting	18	11	2	9	0
Beginner 1	2	Sitting	10	37	20	17	2
Beginner 2	2	Sitting	11	46	26	20	1

**Table 3:** RMSE after reconstruction with different methods (with perfect and algorithmic artifact classifications) for the mocap sequence with a 5% distorted time in the sequence.

	Peaks	Heavy Noise	Step Change	Slow Change
**Distorted**	0.19065	0.17717	0.18069	0.10129
**Linear interpolation (perfect)**	0.00136	0.18322	0.15939	0.18795
**Linear interpolation (classified)**	0.03947	0.18514	0.15623	0.29339
**Savitzky–Golay filter (perfect)**	0.01900	0.04963	0.17440	0.10130
**Savitzky–Golay filter (classified)**	0.08159	0.06855	0.17553	0.10173
**Spline interpolation (perfect)**	*0.00025*	0.11041	0.09780	0.10972
**Spline interpolation (classified)**	*0.03429*	0.68692	0.10895	0.19935
**FFNN predictor (perfect)**	0.01841	*0.01953*	*0.01933*	*0.01875*
**FFNN predictor (classified)**	0.03944	*0.04547*	*0.04409*	*0.11000*

**Table 4:** RMSE after reconstruction with different methods (with perfect and algorithmic artifact classifications) for the mocap sequence with a 10% distorted time in the sequence.

	Peaks	Heavy Noise	Step Change	Slow Change
**Distorted**	0.26906	0.26340	0.26282	0.15037
**Linear interpolation (perfect)**	0.00186	0.25487	0.28409	0.27662
**Linear interpolation (classified)**	0.05144	0.26360	0.27618	0.38908
**Savitzky–Golay filter (perfect)**	0.02678	0.07211	0.25361	0.15046
**Savitzky–Golay filter (classified)**	0.08959	0.08895	0.25510	0.15083
**Spline interpolation (perfect)**	*0.00039*	0.14679	0.15207	0.15955
**Spline interpolation (classified)**	*0.04754*	0.95512	0.16358	0.23918
**FFNN predictor (perfect)**	0.02785	*0.02468*	*0.02581*	*0.02612*
**FFNN predictor (classified)**	0.05537	*0.05201*	*0.06349*	*0.14717*

**Table 5:** RMSE after reconstruction with different methods (with perfect and algorithmic artifact classifications) for the mocap sequence with a 20% distorted time in the sequence.

	Peaks	Heavy Noise	Step Change	Slow Change
**Distorted**	0.38228	0.39623	0.39247	0.21676
**Linear interpolation (perfect)**	0.00273	0.44107	0.44015	0.39172
**Linear interpolation (classified)**	0.07007	0.45679	0.45630	0.55692
**Savitzky–Golay filter (perfect)**	0.03880	0.11228	0.37892	0.21704
**Savitzky–Golay filter (classified)**	0.10383	0.16007	0.38120	0.21748
**Spline interpolation (perfect)**	*0.00058*	0.24337	0.28188	0.25274
**Spline interpolation (classified)**	*0.06753*	1.58979	0.37534	0.35679
**FFNN predictor (perfect)**	0.04169	*0.03711*	*0.03972*	*0.03735*
**FFNN predictor (classified)**	0.07903	*0.11433*	*0.10284*	*0.20549*

## Data Availability

Not applicable.

## Abbreviations

The following abbreviations are used in this manuscript:

FFNN	feed-forward neural network
HF	Hampel filter
HML	Human Motion Laboratory
LS	least squares
M3S	moving three sigma
mocap	MOtion CAPture
MSE	mean square error
NARX-NN	nonlinear autoregressive exogenous neural network
NN	neural network
PJAIT	Polish–Japanese Academy of Information Technology
*RMSE*	root mean squared error
